# The effect of aging and emotions on time processing

**DOI:** 10.1007/s40520-023-02563-z

**Published:** 2023-09-23

**Authors:** Pasquale La Malva, Giulia Prete, Adolfo Di Crosta, Irene Ceccato, Nicola Mammarella, Rocco Palumbo, Alberto Di Domenico

**Affiliations:** Department of Psychological, Health and Territorial Sciences, “G. d’Annunzio” University of Chieti­Pescara, 31, Via Dei Vestini, 66100 Chieti, Italy

**Keywords:** Time reproduction, Temporal judgments, Facial emotions, Aging

## Abstract

**Background:**

Time perception is an automatic process that can be influenced by intrinsic and extrinsic factors.

**Aims:**

This study aimed to investigate the effect of age and emotions on the ability to keep track of short suprasecond intervals.

**Methods:**

Younger adults (N = 108, age range: 18–35) and older adults (N = 51, age range: 65–87) were asked to reproduce, bisect, or double the duration of facial stimuli randomly presented for 1500, 3000, and 4500 ms. The experiment included facial stimuli with positive, negative, or neutral expressions.

**Results:**

The participants across age correctly reproduced intervals but overestimated and underestimated them when asked to bisect and double the intervals, respectively. Overall, when faces were presented with a positive or negative expression, an overestimation of time intervals emerged compared to faces with neutral expressions. Emotions had a greater effect on older adults, who showed a greater overestimation of positive facial expressions and an underestimation of sad, but not angry, facial expressions.

**Discussion:**

The results provide evidence that time perception is influenced by age and emotions, with older adults showing a greater effect of emotions on time processing.

**Conclusion:**

The study suggests an interaction among time processing, age, and emotions, highlighting an automatic relationship among these domains, often considered independent.

## Introduction

Time processing is crucial in most everyday activities, even if this ability shows high inter-individual differences [1] following internal and external conditions [2]. Time perception ranges from milliseconds to centuries, and this particular feature makes it even more difficult to fully understand this cognitive domain and its neural substrate [3]. About forty years ago, Gibbon proposed the Scalar Expectancy Theory, SET [4], to integrate cognitive and psychophysical timing properties in a comprehensive model. The author proposed three stages of time processing. In the first stage, three components play a pivotal role: the clock, constituted by an internal pacemaker emitting pulses; a counter responsible for receiving the pulses and monitoring time; and an attention-controlled switch that allows the pulses to reach the counter. In the second stage, memory is responsible for storing the information processed and, therefore, the subjective experience of time. Finally, in the third stage, the decision selects the appropriate outcome. This pioneering model has also been integrated with more recent evidence about the cerebral bases of time processing, highlighting—for instance—the role of the striatum and the pallidum as two hubs for the early representation of time [5, 6], representing the counter in the SET model. The prefrontal cortex has been identified as responsible for the discrimination between internal and external information [7] and the raw representation of the time interval duration [8]. Finally, parietal and temporal areas are recruited in measuring and storing time (memory stage [9, 10]). Interestingly, a further authoritative model suggests that magnitude, including time, space, and number representations [11], is based on a shared substrate. This involves a frontoparietal network, and there is considerable evidence to support this model at both a behavioral and cerebral level using various paradigms and tasks [10, 12–15]. The evidence indicates that duration estimates can differ depending on task demands [16]. According to this model, there is a left-to-right mental representation that corresponds to a small-to-large distribution of quantities [11, 15, 17]. This representation is known as the Mental Number Line (MNL) or Mental Time Line (MTL), depending on the input being processed [18]. Of note, in addition to magnitude, a left-to-right spatial distribution has also been proposed for more complex features of the perceived stimuli, including emotional valence. It has been shown, for instance, that neither emotional valence nor emotional intensity alone is spatialized in a left-to-right mental line but that they interact with each other in our spatialized mental representation of the stimuli [14].

A robust corpus of evidence demonstrated the impact of emotion on time perception [19]. When we experience emotions, our perception of time is distorted, either as overestimation or underestimation, by the valence and intensity of the affective state and the specific emotion elicited [16, 20]. This effect may happen unconsciously [21] and is amplified in specific clinical populations [22]. Several studies used emotional faces to examine the impact of emotions on time perception in healthy adults, asking them to judge the duration of the stimuli. Results generally reveal that, compared to neutral faces, emotional faces are perceived as lasting longer than actual [23]. Some research found the opposite effect, with emotional stimuli producing an underestimation of the time passed [24]. Emotional faces are often used in studies based on emotions for their specific and unmistakable expressive features for accurate emotional encoding [25, 26]. Beyond faces, researchers adopted a variety of emotional stimuli, such as words [27], sounds [28], images [29], and videos [30, 31], and even tested mood-induction paradigms [32]. Further studies have shown that also experiences and events with significant emotional arousal can modulate psychological well-being by affecting time perception and perspective [33–35]. Overall, findings suggest that emotional experiences and stimuli tend to produce a temporal distortion, compared to neutral stimuli and events, even if a firm consensus on the direction of this distortion has not been reached yet [19].

The impact of emotions on temporal judgments has mostly been explained within the SET framework, suggesting that emotional experiences accelerate the pacemaker and the number of pulses counted, leading to an *overestimation* of the stimuli duration. Other models postulated an attentional mechanism underlying the distortion in temporal judgments, suggesting that emotional stimuli and experiences may “distract” from processing the passage of time [36, 37]. In this case, people *underestimate* the duration of time intervals, as the switch—which allows the time pulses to enter the counter—does not work properly, and some pulses are missed [24].

Notably, the impact of emotions on cognition is magnified in aging: older people are more susceptible to emotional contents in various situations and tasks, such as perception, memory, face recognition, and decision-making [38–41]. Several theoretical models explain the fundamental role of emotions and motivation in older people’s functioning [42–45]. For instance, according to the Selective optimization with compensation model (SOC) and following integrations [46, 47], growing older people are forced to cope with losses in various areas of functioning. To this end, they optimize the available resources and strive toward selected goals. When this is not enough, compensatory strategies are adopted. Specifically, emotion regulation processes are often used as compensatory strategy and this explain why, in face of reduced functioning, older adults report high level of well-being. An alternative model is the Socio-Emotional Selectivity Theory (SST) [48], according to which the future time horizon shapes individual’s goals. When time is perceived as limited, people are oriented toward positive emotional experiences, and avoid negative affects. This is what usually happens in aging, as time is usually perceived as limited in this phase of life. Hence, older people pursue emotional well-being, select positive social relationships, and focus on positive (and avoid negative) stimuli.

Regarding time perception in aging, evidence showed that this ability declines with age, primarily due to the impairments in executive functions, especially working memory, and the changes in the neural circuits underlying time processing [49, 50]. Previous studies demonstrated that older people had shorter time estimates compared to younger adults [51], and showed greater temporal variability in their time judgments [52]. Age-related differences emerged also in related domains involving time perception, such as temporal source memory [53, 54] and subjective time perception [55]. The age-related decline in temporal processing has been confirmed using different temporal judgment tasks, including discrimination, production, and reproduction of time intervals [56]. For instance, studies assessing age-related differences in rhythm monitoring and reproduction (e.g., asking participants to tap at fixed rates) showed that older adults were slower and more variable than younger adults [57]. Difficulties were especially evident in reproduction tasks since they burden the memory component of time estimation [58].

**There are no sources in the current document.**

nce stimulus: 400 ms) or long (reference stimulus: 1600 ms). The authors found that older, but not younger, adults overestimated happy faces compared to neutral faces. Both younger and older participants overestimated anger faces compared to neutral ones, with no differences between the two age groups. Sad stimuli did not produce a significant distortion in temporal judgments. The authors concluded that the positivity effect of aging extends to time perception. The overestimation of angry faces in older adults was confirmed in different studies [60]. Still, other evidence suggested an overestimation for both angry and happy faces compared to neutral ones, in both younger and older adults, without difference between the two age groups [61]. Furthermore, a significant effect of the age of the face also emerged, suggesting that temporal overestimation was greater when seeing older adults’ emotional faces.

## The present study

Starting from the empirical evidence reviewed above, in the current work, we aimed to investigate the possible relationship among temporal judgments, emotions, and aging. Notably, studies showed that the features of the temporal judgment task influence the magnitude of time distortion [19, 62]. Especially when examining the effect of emotions on time perception, the length of the stimuli seems to play a fundamental role [63]. Indeed, as the autonomic response generated by emotional stimuli happens within a few seconds, for a longer duration, other mechanisms are likely to occur in temporal judgments [20]. Furthermore, temporal tasks vary in their requests: in *estimation* tasks, participants are requested to judge the duration of a stimulus, producing a verbal label or answering on a Likert scale; in *discrimination* tasks, participants are asked to compare the stimulus with a standard duration previously learned; in *reproduction* tasks, participants have to actively replicate the duration just experienced [19]. In a recent study, we investigated time processing by exploiting a peculiar set of reproduction tasks, consisting of (1) reproduction of a given time interval, (2) doubling of a given time interval, and (3) bisection of a given time interval [64]. Results confirmed a correct performance in the reproduction task of suprasecond (1500–5000 ms) time intervals, overestimation when time intervals had to be bisected, and underestimation when they had to be doubled. This pattern of results was interpreted as a kind of perceptual aftereffect, in which, after being “subliminally adapted” to the target time interval retained in memory, participants showed an aftereffect similar to those observed in perceptual tasks [65, 66], i.e., enlarging the duration retained in memory when it was relatively shorter, and shortening the same duration when it was relatively longer [64]. Starting from this frame, we wanted to investigate the relationship between time perception and emotions. In particular, we exploited the same paradigm (Reproduction, Bisection, and Doubling tasks), but instead of presenting neutral geometrical shapes for a target interval [64], we presented faces with different emotional valence. While positive valence usually overlaps with the emotion of happiness, several studies pointed out that discrete emotions sharing negative valence should not be considered all the same, as they satisfy different functions and activate different mechanisms [67, 68]. Therefore, in this study, we decided to exploit anger and sadness as negative emotions, as they may differ in various dimensions (e.g., arousal, approach vs. withdrawn motivation) and show specific trajectories across the lifespan [69].

For neutral faces, we hypothesized a similar pattern of results as that found by Momi et al. [64]. Indeed, as we considered neutral faces comparable to the geometric shapes used by Momi and colleagues, we expected a correct performance in the Reproduction condition, and underestimation and overestimation of time in the Doubling and Bisection conditions respectively. We expected an overestimation for positive expressions, and we had two contrasting expectations for negative emotions. Starting from the conflicting literature reviewed above, for negative expressions we would expect either an overestimation (at least for anger) [59, 60], or an underestimation (for both anger and sadness) [14]. This latter hypothesis has also a cerebral base. In fact, a wide literature suggests a left-hemispheric superiority for positive valence and a right-hemispheric superiority for negative valence [70–72], and due to the contralateral organization of the nervous system, the left- vs. right-hemispheric activity should lead a shifting throw the contralateral hemispace when a time interval must be estimated, lengthening or shortening it respectively, according to a MNL/MTL [15].

Finally, we aimed to integrate the study of time perception in aging by considering the role of emotions. We adopted the SST framework and administered the same temporal tasks to younger and older adults. Notably, while previous research adopting reproduction tasks revealed impaired performances in older adults [e.g., 58], to our knowledge, no study has considered the effect of emotions in this type of temporal task. Moreover, no research has analyzed age-related differences in more sophisticated reproduction tasks, such as Bisection and Doubling. Here, we speculated that age-related differences in these tasks might be magnified due to their additional load on working memory (i.e., the second step in the SET model [4]).

## Material and methods

### Participants

The study was carried out on 195 participants: 121 constituted the group of Younger Adults (YA), aged between 19 and 35 (mean ± standard error: 23.98 ± 0.31 years-old; 61 females and 60 males), and 74 constituted the group of Older Adults (OA), aged between 65 and 87 (72.46 ± 0.65 years-old; 39 females and 35 males). All participants signed an informed consent before taking part in the study approved by the local ethical committee, and they self-reported normal or corrected to normal vision and absence of neurological and/or psychiatric conditions. The cognitive functioning of the OA group was evaluated with the Mini-Mental State Examination [73]: the mean score of the sample was 27.85 (± 3.26), ranging between 26 and 30, ensuring the absence of cognitive impairments.

The initial sample was randomly assigned to one of two tasks, differing from one another only for the negative emotion used in the experimental procedure (more details in the Procedure section): 99 participants constituted the subgroup Anger, and the remaining 96 participants constituted the subgroup Sadness. Participants with an overall performance two standard deviations lower or higher than the mean were excluded from the analysis. Statistical analyses were thus carried out on a final sample of 108 YA and 51 OA participants (see Table 1 for details of the final subgroups).

**Table 1:** Demographic information of the four subsamples included in statistical analyses: mean and standard error (M ± SE) of the age; percentage of female participants; M ± SE obtained at the Mini-Mental State Examination (MMSE) for the group OA

	YA-Anger (N = 63)	OA-Anger (N = 25)	YA-Sadness (N = 45)	OA-Sadness (N = 26)
Age (M ± SE)	25.24 ± 0.48	70.64 ± 0.89	22.49 ± 0.3	73.96 ± 1.13
% females	55%	44%	53%	58%
MMSE (M ± SE)		28 ± 0.33		27.73 ± 0.27

### Stimuli

Faces taken from the FACES Life span Database of Facial Expressions [74] were used as stimuli: 9 female and 9 male Caucasian faces were extracted from the database, belonging to the middle-aged group of the FACES (age range: 39–55). For each of the 18 identities, four different facial expressions were selected (i.e., angry, sad, happy, and neutral) for a total of 72 stimuli.

### Procedure

**Fig. 1:**
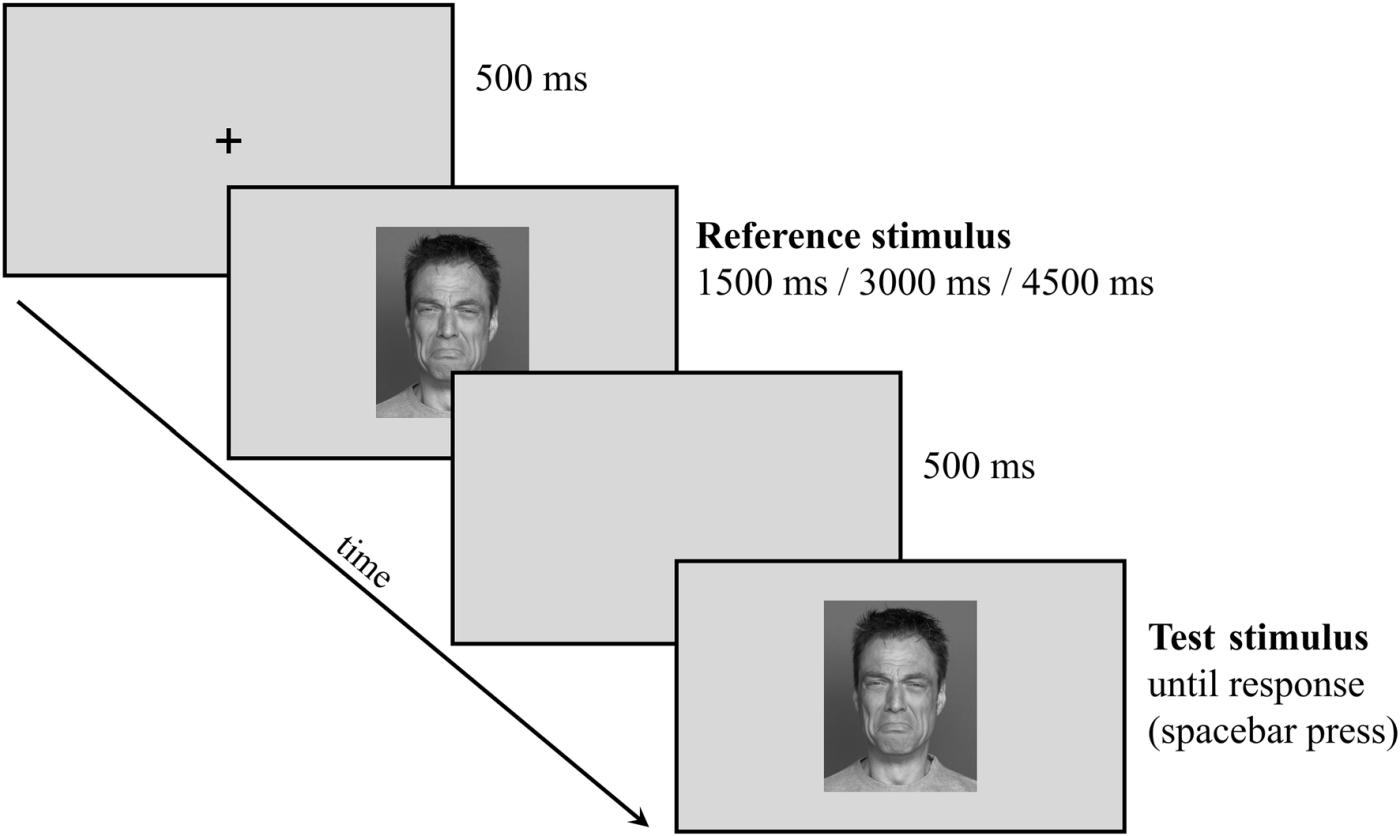
Schematic representation of the experimental procedure: during the task, participants were presented with a face constituting the Reference stimulus whose duration was randomized between 1500 ms, 3000 ms, and 4500 ms; they were asked to press the spacebar when the Test stimulus duration reached either the same duration of the reference stimulus (Reproduction condition), half of the duration of the reference stimulus (Bisection condition), or twice the duration of the Reference stimulus (Doubling condition)

A similar procedure as that used by Momi et al. [64] was exploited in the present study, but emotional valence was added as a parametric factor. Therefore, facial stimuli with emotional (positive vs. negative) and neutral expressions were presented, and participants were asked to estimate their duration. Two versions of the experiment were created, differing in the type of negative facial expressions (i.e., anger vs. sadness). Each trial started with a fixation cross presented in the center of the screen for 500 ms, followed by a facial stimulus (i.e., the reference stimulus), which randomly varied between 1500 ms, 3000 ms, and 4500 ms. Then, after 500 ms of delay, the same facial stimulus appeared, and the participant was required to press the spacebar to indicate the duration of the reference stimulus. In particular, all participants carried out three separate blocks, with random presentation orders, differing from one another for the specific instructions: they were required to press the spacebar when the second stimulus duration reached either the same (Simple Reproduction), the half (Bisection), or twice (Doubling) the reference stimulus duration (see Fig. 1). Participants were told not to count during the stimulus presentation and response phases [75].

A set of 54 trials for each of the three task conditions (Reproduction, Bisection, and Doubling) was administered to each participant for a total of 162 trials. In each task condition, 18 trials contained the positive emotional expression (happiness), 18 trials contained the neutral expression, and the remaining 18 trials contained the negative emotional expression (anger or sadness). Positive, neutral, and negative facial expressions were presented randomly in each block, and their duration was balanced across the three time intervals (1500, 3000, and 4500 ms). The experimental procedure was administered using E-prime 2.0 software (Psychology Software Tools Inc.; www.pstnet.com/eprime) on a Windows laptop PC.

### Statistical analyses

Statistical analyses were performed using Statistica 8.0 software (StatSoft. Inc., Tulsa, USA). The dependent variable was the error in time estimation, measured as the difference between the perceived duration reproduced by the participant and the real duration of the reference stimulus. As in Momi et al. [64], this difference was normalized by computing a T-corrected score (Tc) [76, 77] with the following formula:

$$\mathrm{T-corrected}=\frac{\mathrm{T\,estimated}-\mathrm{ T\,standard }}{\mathrm{T\,standard}}$$

where T estimated is the mean estimation provided by the participant for a given reference stimulus duration (T standard). After this normalization, negative values indicate that the test stimulus is reproduced shorter than the real duration (underestimation), while positive values indicate that the test stimulus is reproduced longer than the real duration (overestimation).

First, we conducted single-sample *t*-test to assess the size of the temporal distortion separately in each task condition (Reproduction, Bisection, and Doubling). Pearson’s correlations were performed to test the relationship between individual performance across Bisection, Reproduction, and Doubling conditions in the whole sample. Finally, a 2 (Age) × 2 (Group) × 3 (Condition) × 3 (Valence) repeated measure Analysis of Variance (ANOVA) was performed. Age and Group were between-subjects factors, while Condition and Valence were within-subjects factors. Tc scores were entered as dependent variable and Duncan tests were used for post-hoc comparisons (*p* < 0.05).

## Results

**Fig. 2:**
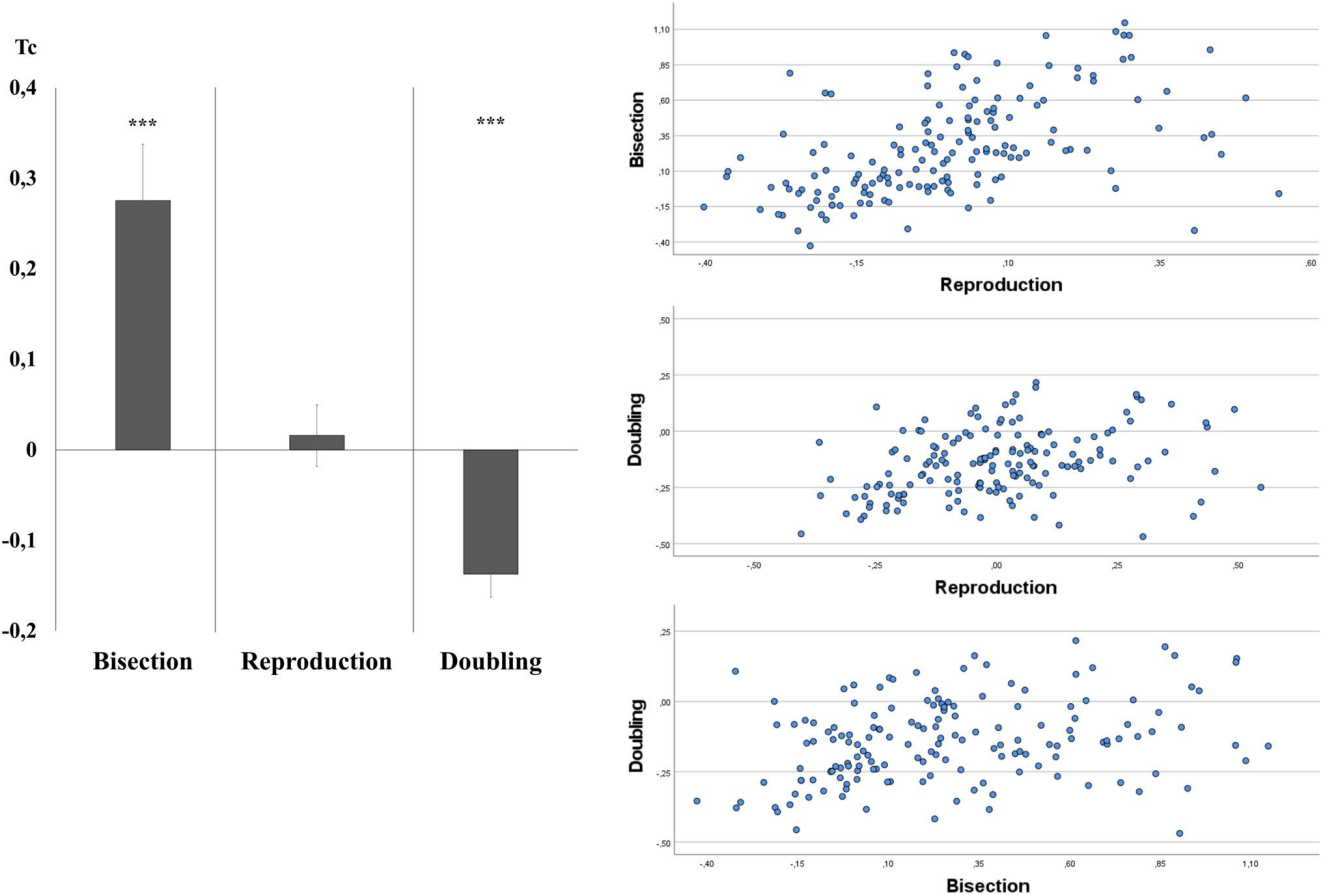
T-corrected measured in each Condition: **a** the Tc scores display a significant overestimation for Bisection and a significant underestimation for Doubling; Reproduction not differing from 0 (bars represent standard errors); **b** scatter plots show significant correlations between Reproduction and Bisection (upper panel), Reproduction and Doubling (central panel), and Bisection and Doubling (bottom panel)

In the first step of the analysis, three single-sample *t*-tests were carried out, comparing the Tc value for each task condition in the whole sample with the correct performance corresponding to Tc = 0 (see Fig. 2a). Exactly in line with the previous evidence [64], results confirmed that Tc did not differ from 0 in the Reproduction condition (*t*_(158)_ = 0.067, *p* = 0.947). Tc was significantly higher than 0 in the Bisection condition (*t*_(158)_ = 9.69, *p* < 0.001), reflecting an overestimation of stimuli duration. Tc was significantly lower than 0 in the Doubling condition (*t*_(158)_ = − 12.319, *p* < 0.001), reflecting an underestimation of stimuli duration.

Results from Pearson’s correlations confirmed that the performances in the three conditions were correlated to each other (Reproduction vs. Bisection: *R*^2^ = 0.27, *p* < 0.001; Reproduction vs. Doubling: *R*^2^ = 0.11, *p* < 0.001; Bisection vs. Doubling: *R*^2^ = 0.09, *p* < 0.001), in line with previous evidence [64] (Fig. 2b).

Finally, to fully investigate the possible effect of each factor and their interactions, we carried out a repeated measure ANOVA entering Age (YA, OA), Group (Anger, Sadness), Condition (Bisection, Reproduction, Doubling) and Valence (Positive, Neutral, Negative) as factors (Table 2).

**Table 2:** Means (and standard errors) of Tc scores in the three task conditions separated by age, group, and valence

	YA-Anger (N = 63)	OA-Anger (N = 25)	YA-Sadness (N = 45)	OA-Sadness (N = 26)
Bisection				
Negative	0.266 (0.040)	0.362 (0.064)	0.293 (0.055)	0.178 (0.088)
Neutral	0.250 (0.044)	0.312 (0.060)	0.130 (0.051)	0.209 (0.082)
Positive	0.343 (0.048)	0.458 (0.072)	0.147 (0.054)	0.355 (0.087)
Reproduction				
Negative	− 0.052 (0.021)	0.022 (0.034)	0.029 (0.029)	0.048 (0.043)
Neutral	− 0.063 (0.025)	0.030 (0.039)	0.006 (0.030)	0.072 (0.043)
Positive	− 0.035 (0.026)	0.034 (0.044)	0.027 (0.033)	0.071 (0.041)
Doubling				
Negative	− 0.176 (0.021)	− 0.080 (0.031)	− 0.119 (0.022)	− 0.316 (0.028)
Neutral	− 0.159 (0.018)	− 0.059 (0.036)	− 0.135 (0.024)	− 0.148 (0.026)
Positive	− 0.147 (0.019)	− 0.072 (0.031)	− 0.133 (0.024)	− 0.108 (0.020)

The main effect of the Condition was significant (*F*_(2, 310)_ = 155.9, *p* < 0.001,* η*_*p*_^*2*^ = 0.50), and post-hoc comparisons confirmed that Tc was higher (overestimation) for Bisection compared to both Reproduction and Doubling and that it was lower (underestimation) for Doubling than for Reproduction (*p* < 0.001 for all comparisons). The significant main effect of Valence (*F*_(2, 310)_ = 21.09, *p* < 0.001,* η*_*p*_^*2*^ = 0.12) showed that time was overestimated (higher Tc) with positive than neutral and negative emotions (*p* < 0.001 for both comparisons), without significant difference between neutral and negative valence. The interaction between Condition and Valence was significant (*F*_(4, 620)_ = 10.8, *p* < 0.001, *η*_*p*_^*2*^ = 0.07), confirming that within each valence, Tc was higher for Bisection and lower for Doubling, compared to Reproduction (all comparisons: *p* < 0.001: Bisection > Reproduction > Doubling). Both in Bisection and in Doubling, Tc was lower (underestimation) for negative than for positive valence; in Bisection, it was higher (overestimation) for negative than for neutral valence (Bisection: positive > negative > neutral), whereas, in Doubling, it was higher for neutral than for negative valence (Doubling: positive and neutral > negative; all comparisons: *p* < 0.01). Importantly, any valence difference emerged in Reproduction. Group significantly interacted with Condition (*F*_(2, 310)_ = 6.2, *p* = 0.002,* η*_*p*_^*2*^ = 0.04), confirming that in both Anger and Sadness subgroups, Tc in Reproduction was significantly lower than in Bisection, and it was higher than in Doubling (all comparisons: *p* < 0.001); moreover, in Bisection, Tc was higher for Anger than for Sadness group (*p* = 0.009).

The main effect of Age was not significant, but it interacted with Valence (*F*_(2, 310)_ = 22.21, *p* < 0.001,* η*_*p*_^*2*^ = 0.13). Post-hoc comparisons revealed that for younger adults, Tc was lower for neutral than for both positive (*p* = 0.004) and negative (*p* = 0.005) emotions, whereas, for OA, Tc was higher for positive compared to neutral and negative emotions (*p* < 0.001), and for neutral compared to negative emotions (*p* = 0.002). Moreover, there was a significant age difference only for positive emotions, with higher Tc for OA compared to YA (*p* = 0.009).

Also, the three-way interaction among Age, Valence, and Group was significant (*F*_(2, 310)_ = 23.27, *p* < 0.001,* η*_*p*_^*2*^ = 0.13). Post-hoc comparisons showed that for older people, in the Sadness group, negative stimuli were underestimated more (lower Tc) than in the Anger group (*p* = 0.012). In all subgroups, positive valence received higher Tc (overestimation) than both neutral and negative valence (all comparisons: *p* < 0.01), except for the YA-Sadness subgroup in which Tc was higher for the negative compared to both neutral and positive valence (*p* < 0.001).

Condition, Valence, and Group significantly interacted (*F*_(4, 620)_ = 4.27, *p* = 0.002,* η*_*p*_^*2*^ = 0.03): post-hoc comparisons confirmed the abovementioned interactions and revealed a group difference in Bisection with neutral and positive valence in which Tc was higher (stronger overestimation) for Anger than for Sadness subgroup (*p* < 0.05). Condition and Valence also interacted with Age (*F*_(4, 620)_ = 5.49, *η*_*p*_^*2*^ = 0.03, *p* < 0.001), and post-hoc comparisons also revealed that only in the Bisection condition and with positive valence, Tc was higher for OA than for YA (*p* = 0.002), showing a stronger overestimation with age for positive emotions.

**Fig. 3:**
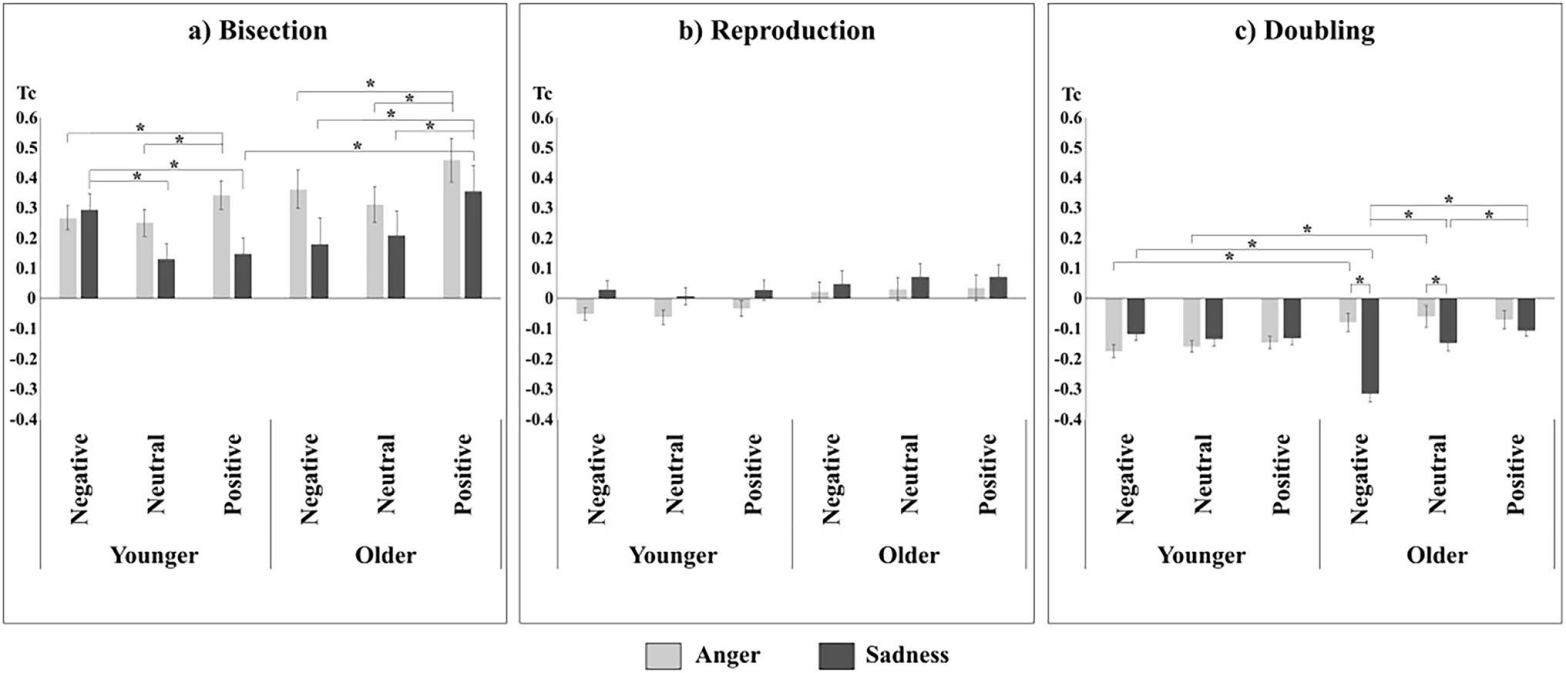
Interaction among Age, Group, and Valence for each Condition: Tc scores in each level of each factor divided for each task: **a** Bisection, **b** Reproduction, **c** Doubling. Bars represent standard errors and asterisks show significant comparisons

Finally, the four-way interaction among Age, Group, Valence, and Condition was significant (*F*_(4, 620)_ = 4.03, *p* = 0.003,* η*_*p*_^*2*^ = 0.03). To better understand the post-hoc comparisons, three separate ANOVAs were carried out, one for each level of Condition (Bisection, Reproduction, Doubling), considering Age and Group as between-subject factors and Valence as within-subject factor. In Bisection, the interaction among Age, Group, and Valence was significant (*F*_(2, 310)_ = 10.15, *p* < 0.001,* η*_*p*_^*2*^ = 0.06; see Fig. 3a). Post-hoc comparisons showed that in the Sadness group, OA overestimated time intervals compared to YA, with positive valence (*p* = 0.045); moreover, YA overestimated time intervals for negative compared to neutral and positive valence (for both comparisons *p* < 0.001), whereas OA overestimated time intervals for positive compared to neutral and negative valence (both *p* < 0.001). In the Anger group, the time overestimation for positive than for neutral and negative valence was significant for both YA (*p* < 0.001 and *p* = 0.005, respectively) and OA (*p* = 0.016 and *p* = 0.044, respectively). No age differences emerged for the Anger group in this task.

The same three-way interaction was not significant in Reproduction (*F*_(2, 310)_ = 0.27, *p* = 0.76; Fig. 3b), but it was significant in Doubling (*F*_(2, 310)_ = 27.67, *p* < 0.001,* η*_*p*_^*2*^ = 0.15; see Fig. 3c). Post-hoc tests showed higher Tc for OA in the Anger than in the Sadness group, both for neutral (*p* = 0.042) and for negative valence (*p* < 0.001). Furthermore, in the Anger group, YA compared to OA underestimated time intervals for neutral (*p* = 0.021) and negative stimuli (*p* = 0.026). On the contrary, in the Sadness group, OA underestimated time intervals compared to YA for negative valence (*p* < 0.001), with no further differences between age groups; moreover, OA in the Sadness group underestimated time intervals with negative compared to neutral and positive valence (both *p* < 0.001), and with neutral than with positive valence (*p* = 0.032). No other difference emerged.

## Discussion

The results fully confirmed those previously found using neutral geometrical shapes: participants correctly reproduce suprasecond time intervals. However, they showed a significant overestimation vs. underestimation when intervals must be bisected vs. doubled, respectively [64]. We confirmed that the performance in the three tasks (Reproduction, Bisection, and Doubling) correlated to each other, thus providing support for the SET theory [4] and for an internal clock keeping track of time. As Momi et al. [64], the present results confirmed that when participants were asked to manipulate time mentally, they overestimated/underestimated time intervals when they should mentally bisect/double their durations, respectively, extending this evidence also to older adults. We can conclude that the convergence of performance towards a middle interval is similar to aftereffects observed in perceptual tasks [64, 65, 78]. It is important to underline that a similar pattern has also been observed in some temporal discrimination tasks where participants had to decide if the second interval was shorter or longer than the first one (e.g., [78, 79]). After being adapted to a long/short reference stimulus, the following test stimulus is judged as shorter/longer than the first one, respectively [10, 78]. Similar biases have been observed in other cognitive domains, such as space perception [80], which shares neural substrates with time representation [11]. Our brain may fail to objectively compute time due to internal and external factors affecting the processing of a given interval [81]. However, our brain seems to be able to adapt and counterbalance accordingly based on the situation [82].

Here we showed that such compensation could also be affected by the emotional valence of the stimuli. Our findings revealed a significant effect of emotions on time perception: we found that positive facial expressions led to an overestimation of time intervals, confirming a link between two domains which theoretically must be quite different from one another, namely objective time tracking and emotional processing of the stimuli [59]. Current findings contribute to the ongoing debate on time perception, supporting the view that emotions influence temporal judgments and that the nature of the task is crucial in highlighting the effects of emotional valence [19]. Indeed, we found no time distortion for emotional stimuli in the Reproduction task, in line with previous studies [62], but over/underestimation emerged when participants were involved in more complex temporal judgments.

Furthermore, current results are crucial to support a strict relationship between time and emotional processing, providing further evidence for an intrinsic web connecting cognitive domains that apparently are so different from one another. This view aligns with previous evidence suggesting, for instance, that space and emotions are related [14], enlarging this mutual relation to timing. The fact that positive, but not negative, valence is linked to an overestimation seems to support the speculation of a left-hemispheric activity during the perception of positive emotions [14, 72, 83, 84], which would lead to a rightward shift on the MTL, and thus causing an overestimation. Moreover, this pattern of results also corroborates previous evidence showing an overestimation of positive emotions across ages [61]. Importantly, intriguing age-related effects emerged from our study: across tasks, younger participants overestimated time intervals with both positive and negative stimuli compared to neutral, whereas older participants overestimated time intervals with positive stimuli and underestimated negative stimuli compared to the neutral ones. Moreover, older adults, compared to younger adults, overestimated the time when happy faces were presented, in particular during the Bisection task (in which time overestimation is evident in the whole sample), revealing a selective effect of happiness in aging. This result is in line with the Socio-Emotional Selectivity Theory and reveals a positivity bias also in time perception [38, 41]. Present results closely match those obtained by Nicol et al. [59] who used a temporal discrimination task and found that older adults overestimated happy faces, while no age-related differences appeared for angry and sad stimuli.

The three-way interaction, including the difference between anger and sadness, further confirmed the sensitivity of older participants to specific emotions: in this age group an underestimation emerged for sadness but not for anger, compared to neutral stimuli. For younger participants, anger and sadness did not differ, even if sad but not angry faces were considered to last longer than neutral ones, showing a trend opposite to that found in older adults. This pattern of results was supported by the interaction involving all the factors considered in this study: while no significant interaction emerged among age, valence, and emotion in the Reproduction task, interesting age-related differences appeared in the Bisection and Doubling tasks as a function of the negative emotion considered. In the Bisection task, older adults overestimated positive stimuli over neutral and sad faces, in line with the positivity effect [44, 59]. In comparison, younger adults overestimated sad compared to neutral and positive stimuli. However, older and younger participants behaved similarly for angry stimuli, showing an overestimation of happy faces compared to neutral and angry ones. In the Doubling task, again, only in the Sadness group, interesting effects emerged: while for younger adults, the emotional valence did not impact temporal perception, older people perceived sad stimuli last shorter than neutral stimuli, which in turn were perceived shorter than positive stimuli. Older people perceived sad faces to last almost three times shorter than younger adults. With angry faces, emotional valence did not influence temporal processing in younger or older participants, even if older adults showed lower underestimation compared to younger adults for neutral and angry faces.

Overall, sadness seems to show a specific impact on time perception in different phases of life, while anger showed a less variable effect across ages. These findings echoed those from other cognitive domains, in which sadness and anger showed differential trajectories and effects in aging [69, 85]. Present results may be explained in light of the discrete emotion theory of affective aging—DEA [45]. According to this framework, from an evolutionary perspective, anger and sadness play different functions. While anger is crucial for younger people as it motivates actions and problem-solving, sadness is more salient for older adults as it favors disengagement in a time of unavoidable losses [44]. Regarding time perception, we speculated that older people’s tendency to avoid (underestimate) sadness more than anger could be due to the greater salience of this negative affect. While the frequency of sad experiences increases with age, older adults also showed regulatory mechanisms to cope with these events [86]. Also, from a physiological perspective, preliminary evidence suggested that individuals who disengage more easily when experiencing sadness may show greater emotional well-being in terms of cortisol levels [87]. We also acknowledge that our findings contrast with the Motivational Dimensional Model of Time Perception, recently proposed by Gable et al. [88]. According to this framework, emotions should be considered in light of the actions they elicit, approaching or withdrawing, and the intensity of these motivations. Subsequently, approach motivation should hasten time perception (i.e., time underestimation), while withdrawal motivation should slow temporal judgments (i.e., time overestimation). Our results seem to point in the opposite direction: happiness and anger (approach-motivated emotions) produced an overestimation. Sadness, considered an approach-motivated emotion by Gable and colleagues, led to time underestimation, at least in older adults. However, other researchers consider sadness a withdrawn-motivated emotion. Thus, the picture is not well-defined.

The present results should be interpreted considering some caveats of the study. First, we focused on happiness, anger, and sadness. Therefore, our conclusions are limited to these three discrete emotions. It is likely that other emotions affect time perception in different ways [89] and that valence (positive vs. negative) is insufficient to predict the direction of the temporal distortions. Second, we used facial expressions to convey emotional valence. Noteworthy, faces are often used as experimental stimuli for their naturalistic validity representing a core element of the perception and experience of emotions, among other features [90–94]. Nevertheless, future works may extend current findings to other emotional stimuli, such as sounds (e.g., voices or music), and analyze mood induction effects of the time perception of neutral stimuli.

To summarize, we extended the previous literature on time perception by a) adopting an innovative, multifaceted temporal reproduction task; b) analyzing age-related differences; and c) exploring the role of emotional valence. First, we confirmed that young and older adults showed similar temporal distortions when involved in sophisticated temporal judgment tasks: our results revealed that time intervals to be doubled are perceived shorter than actual, and time intervals to be bisected are perceived longer than actual. Second, current results confirmed that emotions generate selective time distortions in young and older adults. Overall, both positive and negative emotions in younger adults lead to overestimating time intervals. In older adults, positive stimuli were perceived longer than neutral, while negative stimuli were perceived as shorter than neutral, confirming the presence of a positivity effect in aging. We conclude that time perception and emotions are two distinct cognitive domains, but they influence each other so that one dimension predictably affects the other. Furthermore, we showed that each discrete emotion could produce a specific effect on time estimation and that such effects change across ages. Further studies are needed to elucidate this relationship. Still, the present results revealed that time estimation is a relatively simple task that could be useful to shed light on the complex link among different cognitive domains at different ages. On a practical point of view, current findings align and extend previous studies revealing age-related impairments in time perception. Notably, researchers are suggesting that the size of temporal distortions can be used to discriminate between healthy aging and pathological cognitive impairments [95]. Future studies should test whether the impact of emotions on time perception is different in healthy and pathological aging, and whether potential differences could be used as diagnostic tool for early cognitive decline [96].

## Data Availability

The datasets generated and analyzed during the current study are available from the corresponding author upon reasonable request.
